# Association between psoriasis and coronary artery calcification: A systematic review and meta-analysis

**DOI:** 10.3389/fcvm.2022.1044117

**Published:** 2022-11-25

**Authors:** Huihui Wu, Zeyu Luo, Juanhua Liu, Diqing Luo, Luli Song, Yukun Zhao

**Affiliations:** ^1^Department of Dermatology, The East Division of The First Affiliated Hospital, Sun Yat-sen University, Guangzhou, China; ^2^Department of Dermatology, Guangzhou Development District Hospital, Guangzhou, China

**Keywords:** psoriasis, coronary artery calcification, atherosclerosis, inflammation, meta-analysis

## Abstract

**Background:**

Psoriasis and atherosclerosis have overlapping pathophysiological mechanisms. However, the association between psoriasis and coronary artery calcification (CAC), a hallmark of atherosclerosis and a predictor of poor cardiovascular prognosis, remains to be determined. We performed a systematic review and meta-analysis to comprehensively evaluate the association between these related inflammatory conditions.

**Methods:**

Observational studies evaluating the relationship between psoriasis and CAC were retrieved by searching PubMed, Cochrane’s Library, and Embase databases. Presence of CAC was confirmed according to an Agatston’s Score >0 upon computed tomography examination. A random-effect model incorporating between-study heterogeneity was used to pool the results.

**Results:**

Sixteen studies involving 3,039 patients with psoriasis and 46,191 controls without psoriasis were included in the meta-analysis. All participants were without previously known cardiovascular diseases. Pooled results showed that psoriasis was associated with overall CAC [odds ratio (OR): 1.54, 95% confidence interval: 1.23–1.91, *p <* 0.001; *I*^2^ = 57%], after matching or adjusting the conventional cardiovascular risk factors. Subgroup analyses showed that study country, comorbidity of psoriatic arthritis, baseline Psoriasis Area and Severity Index, and duration of psoriasis (*p* for subgroup difference all >0.05) did not significantly affect the association of psoriasis and CAC. However, a stronger association was observed in younger patients (mean age <50 years, OR: 2.63, *p* < 0.001) compared to older patients (≥50 years, OR: 1.24, *p* = 0.02; *p* for subgroup difference <0.001).

**Conclusion:**

Psoriasis is associated with CAC, and the association may be stronger in younger patients.

## Introduction

Psoriasis is an autoimmune and inflammatory skin disorder that primarily affects the elbows, knees, scalp, umbilicus, and lower back, with sharply defined erythematous plaques covered in silvery-white scales (1). The prevalence of psoriasis, according to peer-reviewed literature, varies from 1 to 9% in the adult population across different countries (2, 3). Along with causing localized inflammation of the skin caused by the scaling plaques, psoriasis also promotes systemic inflammation, especially in the vascular system, which may expose the affected inidvidual to a higher risk of cardiovascular events (4, 5). Recent literature suggests that the pathophysiological mechanisms underlying psoriasis may overlap with those underlying the pathogenesis of atherosclerosis, such as vascular inflammation, endothelial dysfunction, and oxidative stress-related injuries (6, 7). Further, a recent meta-analysis including 31 cohort studies showed that patients with psoriasis had higher risks of multiple adverse cardiovascular outcomes, including incidences of myocardial infarction, stroke, cardiac death, ischemic heart disease, thromboembolism, and arrhythmia (8). Therefore, studies are needed to determine the key pathophysiological changes involved in the pathogenesis of cardiovascular diseases in patients with psoriasis.

Accumulating evidence suggests that coronary artery calcification (CAC) is a hallmark of atherosclerosis and a predictor of poor cardiovascular prognosis in the general population (9). Clinically, computed tomography (CT) has become an widely utilized and effective non-invasive tool for assessing CAC (10, 11). A high CAC score independently predicts the risk of adverse cardiovascular events and all-cause mortality in both asymptomatic and symptomatic population without coronary artery disease (CAD) (12–14). Early meta-analyses suggested a potential association of psoriasis and a higher risk of cardiovascular events. One meta-analysis in 2013 involving nine studies showed that severe psoriasis was associated with a 39% increased risk of cardiovascular mortality, 70% increased risk of myocardial infarction, and 56% increased risk of stroke (15). A subsequent meta-analysis further suggested that the risk of myocardial infarction and stroke was increased even in patients with mild psoriasis (16). However, previous studies evaluating the association of psoriasis and CAC have shown inconsistent results (17). Some studies have suggested a significant association between psoriasis and CAC (18–24), while others have not demonstrated an association (25–33). In this study, we performed a systematic review and meta-analysis to comprehensively evaluate the association of psoriasis and CAC.

## Materials and methods

The Preferred Reporting Items for Systematic Reviews and Meta-analyses (PRISM) statement (34, 35) was followed in designing, performing, and reporting the meta-analysis. This systematic review and meta-analysis was conducted in accordance with Cochrane’s Handbook guidelines (36).

### Literature retrieval

The electronic databases PubMed, Cochrane’s Library, and Embase were searched from the inception of the respective databases until June 15, 2022. A combined search term was used, including (1) “calcification” OR “coronary”; and (2) “psoriasis” OR “psoriases” OR “Pustulosis of Palms and Soles” OR “Pustulosis Palmaris et Plantaris” OR “Palmoplantaris Pustulosis” OR “Pustular Psoriasis of Palms and Soles.” The search was limited to human studies published in full-length articles. No restriction was applied regarding the language of publication. To supplement our search, we manually reviewed the citations of relevant original articles and review articles.

### Study selection

A PICOS-based inclusion criterion was used for this study.

P (patients): Adult participants with no previous history of cardiovascular diseases in a clinic or a hospital-based setting.

I (exposure): Patients with a confirmed diagnosis of psoriasis, with or without psoriatic arthritis (PsA). We did not exclude patients with psoriasis of special sites (such as nail psoriasis, scalp psoriasis, and palmoplantar psoriasis) or pustular psoriasis.

C (control): Participants without psoriasis.

O (outcomes): The primary outcome of the meta-analysis was the odds ratio (OR) for overall CAC between patients with psoriasis and controls. The presence of overall CAC was confirmed according to an Agatston’s Score (AS) >0 upon CT examination, as previously defined (37). ORs for moderate to severe CAC (AS > 100) and severe CAC (AS > 400) were evaluated between controls and patients with psoriasis.

S (study design): Observational studies, including cohort studies, case-control studies, and cross-sectional studies were included in the meta-analysis. The meta-analysis excluded reviews, preclinical studies, case reports or case series, studies without psoriasis patients, studies lacking CAC evaluation, and studies without related outcome data.

### Data collection and quality assessment

Two authors separately searched and analyzed the literature, collected data, and assessed study quality. A third author was consulted if discrepancies were encountered. Study information (first author, location, and publication year), study design, psoriasis patient characteristics [sample size, with or without PsA, baseline Psoriasis Area and Severity Index (PASI), and duration of psoriasis], control characteristics, and CAC analysis method and results were collected. Variables that were adjusted or matched when the association between psoriasis and CAC were also recorded. Study quality was assessed using the Newcastle-Ottawa Scale (NOS) (38) based on criteria for participant selection, comparability of groups, and validity of results. Study quality was defined by the number of stars between one and nine, with more stars representing a better study quality.

### Statistical analyses

The association between psoriasis and CAC (overall, moderate-to-severe, and severe CAC) is presented as OR and its corresponding 95% confidence interval (CI). ORs and standard errors were calculated using the 95% CIs or *p*-values, followed by logarithmical transformation. Cochrane’s Q test and *I*^2^ statistics were used to estimate study heterogeneity (39), with significant heterogeneity reflected by an *I*^2^ > 50%. The results were combined using a random-effects model incorporating the influence of heterogeneity (36). An influencing analysis omitting one study at a time was conducted to determine the effect that each study had on the overall results (40). If adequate datasets were available (≥10), subgroup analyses were conducted to examine how study characteristics influenced the results. If ≥10 studies were available for the meta-analysis, an estimate of publication bias was made by constructing funnel plots and applying Egger’s regression asymmetry test to the visual judgment of symmetry (41). Statistical analyses were conducted using RevMan (version 5.1; Cochrane Collaboration, Oxford, UK) and Stata (version 12.0; Stata Corporation, USA).

## Results

### Studies obtained

**FIGURE 1:**
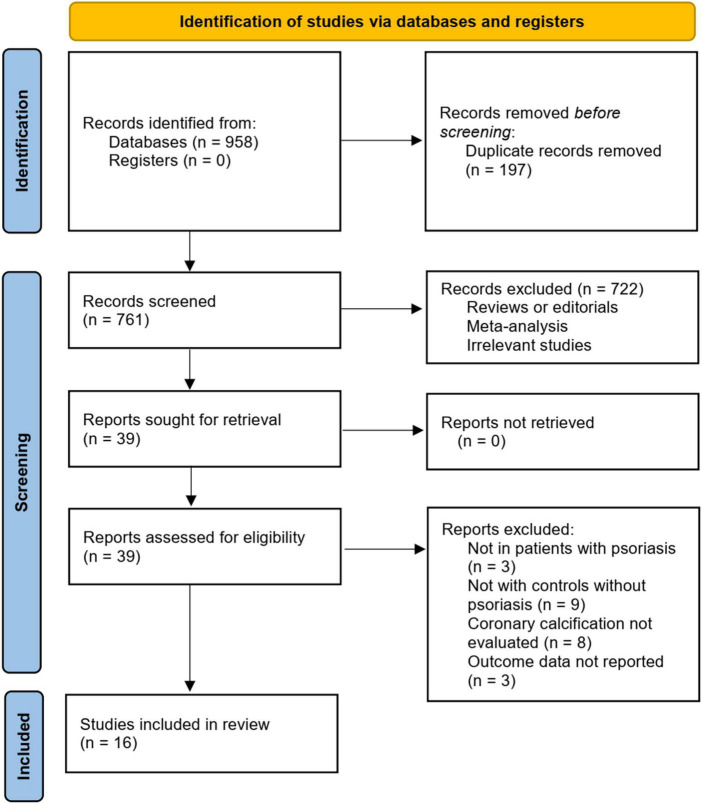
Summary of the literature search and study identification process.

Figure 1 summarizes the literature search procedure. In short, the initial database search retrieved 958 articles. A total of 761 remained after excluding duplicated records. Subsequently, 722 articles were excluded since their titles and abstracts were not relevant to the meta-analysis, leaving 39 studies for full-text review. Finally, after excluding nine studies through full-text review, 16 studies (18–33) were included. The reasons for removing the nine studies are also presented in Figure 1.

### Characteristics of the included studies

As shown in Table 1, fifteen case-control studies (18–23, 25–33) and one cross-sectional study (24), including 3,039 patients with psoriasis and 46,191 controls without psoriasis, were included in this meta-analysis. The studies were published between 2007 and 2022, and conducted in Germany, Brazil, China, Japan, Denmark, Portugal, United States, and Ireland. Adult patients with psoriasis were included as cases in all of the included studies. Two of the studies included psoriasis patients without PsA (19, 20), five studies included psoriasis patients with or without PsA (21, 22, 24, 27, 32), two studies included PsA patients only (23, 31), and the remaining seven studies did not specify whether patients with PsA were included (18, 25, 26, 28–30, 33). The baseline PASI for the patients with psoriasis varied between 1.2 and 14.2, and the duration of psoriasis ranged from 3 to 28 years. Participants without psoriasis or previously diagnosed cardiovascular diseases were included as controls. The mean age of the included participants ranged from 41 to 69 years. For all of the included studies, presence of CAC was evaluated based on AS on CT scans. Traditional cardiovascular risk factors, such as age, sex, family history of cardiovascular diseases, smoking, body mass index (BMI), hypertension, diabetes, and dyslipidemia were adjusted or matched when the association between psoriasis and CAC was evaluated. The NOS scores of the included studies ranged from 8 to 9 stars, suggesting moderate to good study quality (Table 2).

**TABLE 1:** Characteristics of the included studies.

References	Location	Design	Patients with psoriasis	PsA patients	PASI for the patients	Duration of psoriasis (years)	Controls	No. of patients	No. of controls	Mean age (years)	Male (%)	Evaluation of CAC	Variables adjusted or matched
Ludwig et al. (18)	Germany	C-C	Patients with a >10 years history of plaque-type psoriasis but no previous CVD	Not specified	NR	23.1	Non-psoriatic patients with no previous CVD	32	32	49.1	82.2	Agatston’s Score on 16-row spiral CT	Age, race, sex, smoking habits, family history of CVD, DM, HTN, BMI, TC, TG, and CRP
Yiu et al. (19)	China	C-C	Patients with chronic plaque psoriasis but no previous CVD	Excluded	14.2	15.7	Non-psoriatic patients with no previous systemic inflammatory disease or CVD	70	51	45.4	71	Agatston’s Score on 64-slice MDCT	Age, sex, smoking, hypertension, DM, TC, LDL-C, and hsCRP
Staniak et al. (26)	Brazil	C-C	Patients with a >3 years history psoriasis but no previous CVD	Not specified	NR	>3	Non-psoriatic patients with no previous CVD	221	718	55.8	50.3	Agatston’s Score on 64-slice MDCT	Age, sex, FRS, and hsCRP
Balci et al. (25)	Japan	C-C	Patients with a history of systemic or inpatient treatment for psoriasis but no previous CVD	Not specified	9.8	14.9	Non-psoriatic patients with no previous CVD	38	38	41	68.4	Agatston’s Score on 64-slice MDCT	Age, sex, smoking habits, family history of CVD, DM, HTN, BMI, LDL-C, and UA
Hjuler et al. (27)	Denmark	C-C	Patients with severe plaque psoriasis but no previous CVD	Partial	≥10	28.1	Non-psoriatic patients with no previous CVD	58	33	49.2	70.3	Agatston’s Score on non-contract CAC-CT	Age, sex, smoking, family history of CVD, DM, and HTN
Seremet et al. (28)	Turkey	C-C	Patients with severe plaque psoriasis but no previous CVD	Not specified	6.6	16	Non-psoriatic patients with no previous CVD	40	42	41.8	46.3	Agatston’s Score on 128-slice MDCT	Age, race, sex, smoking habits, family history of CVD, DM, HTN, BMI, TC, TG, and hs-CRP
Torres et al. (20)	Portugal	C-C	Patients with severe plaque psoriasis but no previous CVD	Excluded	12.8	21.9	Non-psoriatic patients with no previous CVD	100	202	52.1	64.3	Agatston’s Score on 64-slice MDCT	Age, sex, smoking habits, family history of CVD, DM, HTN, BMI, and TC
Mansouri et al. (21)	USA	C-C	Patients with moderate to severe plaque psoriasis but no previous CVD	Partial	1.2	11	Non-psoriatic patients with no previous CVD	129	129	51.5	49.6	Agatston’s Score on 128-slice MDCT	Age, sex, smoking, LDL-C, HTN, DM, and concurrent medications
Santilli et al. (22)	USA	C-C	Patients with psoriasis but no previous CVD	Partial	10	19.4	Non-psoriatic patients with no previous CVD	207	76	48	54.4	Agatston’s Score on 64-slice MDCT	Age, sex, race, BMI, smoking, HTN, HDL-C, and hsCRP
Shen et al. (23)	China	C-C	Patients with PsA but no previous CVD	All	3.7	6.1	Non-psoriatic patients with no previous CVD	90	240	49.8	58.5	Agatston’s Score on 64-slice MDCT	Age, sex, smoking, BMI, DM, HTN, and dyslipidemia
Lerman et al. (29)	USA	C-C	Patients with plaque psoriasis but no previous CVD	Not specified	5.7	20	Non-psoriatic patients with no previous CVD	105	125	54.7	60	Agatston’s Score on MDCT	Age, sex, smoking, HTN, DM, and dyslipidemia
Szentpetery et al. (31)	Ireland	C-C	Patients with PsA but no previous CVD	All	4.7	18.7	Non-psoriatic patients with no previous CVD	50	50	57.8	51	Agatston’s Score on 64-slice MDCT	Age, sex, smoking, BMI, DM, HDL-C
Momose et al. (30)	Japan	C-C	Patients with moderate to severe psoriasis vulgaris but no previous CVD	Not specified	8.3	13.8	Non-psoriatic patients with no previous CVD	86	31	57.5	64.1	Agatston’s Score on MDCT	Age, BMI, smoking, diabetes, HTN, and LDL-C
Tinggaard et al. (24)	Denmark	CS	Patients with psoriasis but no previous CVD	Partial	NR	NR	Non-psoriatic patients with no previous CVD	1726	44296	57.6	46.8	Agatston’s Score on MDCT	Age, sex, smoking, BMI, CCI, DM, HTN, and dyslipidemia
Ellis et al. (32)	USA	C-C	Patients with severe psoriasis but no previous CVD	Partial	NR	NR	Non-psoriatic patients with no previous rheumatologic condition and CVD	25	16	46.6	46.3	Agatston’s Score on MDCT	Age, sex, smoking habits, family history of CVD, DM, HTN, BMI, dyslipidemia, and hsCRP
Weber et al. (33)	USA	C-C	Patients with psoriasis but no previous CVD	Not specified	NR	NR	Non-psoriatic patients with no previous CVD	62	112	68.9	31	Agatston’s Score on MDCT	Age, sex, BMI, smoking, HTN, DM, and dyslipidemia

PsA, psoriatic arthritis; PASI, Psoriasis Area and Severity Index; C-C, case-control; CS, cross-sectional; CVD, cardiovascular disease; NR, not reported; CAC, coronary artery calcification; MDCT, multi-detector CT; DM, diabetes mellitus; HTN, hypertension; BMI, body mass index; CRP, C-reactive protein; hsCRP, high-sensitivity C-reactive protein; TC, total cholesterol; TG, triglyceride; LDL-C, low-density lipoprotein cholesterol; HDL-C, high-density lipoprotein cholesterol; FRS, framingham risk score; CCI, charlson comorbidity index.

**TABLE 2:** Study quality evaluation using the Newcastle-Ottawa Scale (NOS).

References	Adequate definition of cases	Representativeness of cases	Selection of controls	Definition of controls	Control for age and sex	Control for other confounders	Exposure ascertainment	Same methods for events ascertainment	Non-response rates	Total
Ludwig et al. (18)	1	1	1	1	1	1	1	1	1	9
Yiu et al. (19)	1	1	1	1	1	1	1	1	1	9
Staniak et al. (26)	1	1	1	1	1	1	1	1	1	9
Balci et al. (25)	1	1	1	1	1	1	1	1	1	9
Hjuler et al. (27)	1	1	1	1	1	1	1	1	1	9
Seremet et al. (28)	1	1	1	1	1	1	1	1	1	9
Torres et al. (20)	1	1	1	1	1	1	1	1	1	9
Mansouri et al. (21)	1	1	1	1	1	1	1	1	1	9
Santilli et al. (22)	1	1	1	1	1	1	1	1	1	9
Shen et al. (23)	1	1	1	1	1	1	1	1	1	9
Lerman et al. (29)	1	1	1	1	1	1	1	1	1	9
Szentpetery et al. (31)	1	1	1	1	1	1	1	1	1	9
Momose et al. (30)	1	1	1	1	0	1	1	1	1	8
Tinggaard et al. (24)	1	1	1	1	1	1	1	1	1	9
Ellis et al. (32)	1	1	1	1	1	1	1	1	1	9
Weber et al. (33)	1	1	1	1	1	1	1	1	1	9

### Association between psoriasis and coronary artery calcification

**FIGURE 2:**
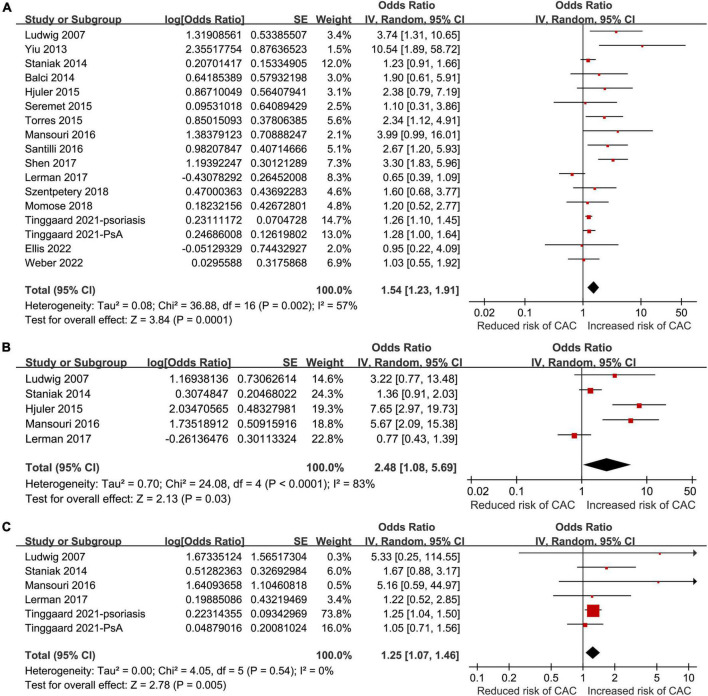
Forest plots of the meta-analyses regarding the association between psoriasis and coronary artery calcification (CAC). **(A)** Meta-analysis of the presence of overall CAC. **(B)** Meta-analysis of the presence of moderate to severe CAC. **(C)** Meta-analysis of the presence of severe CAC.

All of the included studies reported an association between psoriasis and overall CAC. Since one study reported the association in patients with and without PsA separately (24), these two datasets were independently included in the meta-analysis. Therefore, 17 datasets from the 16 studies (18–33) were available to analyze the association between psoriasis and CAC. Pooled results showed that psoriasis was associated with an overall CAC (OR: 1.54, 95% CI: 1.23–1.91, *p* < 0.001; *I^2^* = 57%; Figure 2A), after matching or adjusting for conventional cardiovascular risk factors. Influencing analysis by sequentially excluding one dataset at a time showed consistent results (OR: 1.41–1.65, *p* all <0.05). Sensitivity analyses showed that the association between psoriasis and overall CAC was consistent in the meta-analysis of case-control studies only (OR: 1.74, 95% CI: 1.26–2.42, *p* < 0.001; *I^2^* = 60%) and in the meta-analysis of studies with high quality (NOS nine, OR: 1.56, 95% CI: 1.24–1.97, *p* < 0.001; *I^2^* = 59%; Table 3). Subgroup analyses showed that study characteristics did not significantly affect the association between psoriasis and overall CAC (*p* for subgroup difference all >0.05; Table 3). However, a stronger association was observed in younger patients (mean age <50 years, OR: 2.63, 95% CI: 1.82–3.80, *p* < 0.001; *I^2^* = 8%) than older patients (≥50 years, OR: 1.24, 95% CI: 1.04–1.47, *p* = 0.02; *I^2^* = 35%; Table 3). Additionally, a meta-analyses including five studies showed that psoriasis was also associated with moderate-to-severe CAC (OR: 2.48, 95% CI: 1.08–5.69, *p* = 0.03; *I^2^* = 83%; Figure 2B) and severe CAC (OR: 1.25, 95% CI: 1.07–1.46, *p* = 0.005; *I^2^* = 0%; Figure 2C).

**TABLE 3:** Results of sensitivity and subgroup analyses of the association between psoriasis and coronary artery calcification (CAC).

Study characteristics	Datasets number	OR (95% CI)	*I* ^2^	*P* for subgroup effect	*P* for subgroup difference
Study design					
Only case-control studies	15	1.74 (1.26, 2.42)	60%	NA	<0.001
Study quality					
Only NOS = 9	16	1.56 (1.24, 1.97)	59%	NA	<0.001
Country					
Asian	4	2.53 (1.23, 5.22)	55%	0.01	–
Non-Asian	13	1.35 (1.11, 1.65)	44%	0.003	0.10
Mean age					
<50 years	8	2.63 (1.82, 3.80)	8%	<0.001	–
≥50 years	9	1.24 (1.04, 1.47)	35%	0.02	<0.001
Men					
<54%	8	1.26 (1.13, 1.41)	0%	<0.001	–
≥54%	9	2.16 (1.28, 3.64)	71%	0.004	0.09
Inclusion of PsA					
Psoriasis without PsA	3	2.23 (0.95, 5.25)	76%	0.07	–
Psoriasis or PsA	4	2.39 (1.39, 4.12)	0%	0.002	–
Only PsA	3	1.84 (0.97, 3.50)	76%	0.06	0.83
PASI					
<10	7	1.58 (0.88, 2.81)	69%	0.1	–
≥10	4	2.75 (1.72, 4.39)	0%	<0.001	0.14
Duration of psoriasis					
<18 years	6	2.37 (1.34, 4.18)	43%	0.003	–
≥18 years	6	1.85 (1.01, 3.39)	71%	0.04	0.56

OR, odds ratio; CI, confidence interval; CAC, coronary artery calcification; NOS, Newcastle-Ottawa Scale; PsA, psoriatic arthritis; PASI, Psoriasis Area and Severity Index; NA, not applicable.

### Publication bias

**FIGURE 3:**
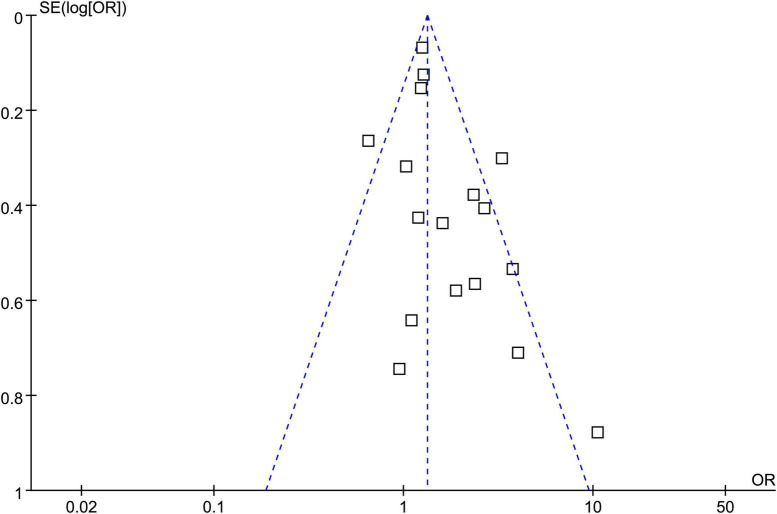
Funnel plots assessing publication bias underlying the meta-analysis of psoriasis and overall coronary artery calcification (CAC).

Funnel plots of the association between psoriasis and CAC show symmetry, indicating low risks of publication bias. Egger’s regression tests showed similar results (*p* = 0.57) (Figure 3).

## Discussion

In this systematic review and meta-analysis, we pooled the results of 16 available case-control and cross-sectional studies, and the results showed that patients with psoriasis were at higher risk of overall CAC compared to controls without psoriasis. Results of influencing analysis showed consistent results. Subgroup analyses showed that study characteristics did not significantly affect the association of psoriasis with CAC; however, there was a stronger association between psoriasis and CAC in younger patients (<50 years) compared to older patients (≥50 years). Finally, a meta-analyses with five studies showed that psoriasis was also associated with moderate-to-severe CAC and severe CAC. Taken together, these findings suggest that psoriasis is associated with CAC, and the association may be stronger in younger patients. The presence of CAC may be one of the early and key pathological changes underlying the pathogenesis of cardiovascular diseases in patients with psoriasis.

To the best of our knowledge, few meta-analyses have been performed to evaluate potential subclinical atherosclerosis in patients with psoriasis. An early meta-analysis including 16 case-control studies showed that patients with PsA had an increase in common carotid artery intima-media thickness (cIMT), a higher frequency of carotid plaques, and a lower flow-mediated dilation (FMD), indicating endothelial dysfunction and systematic atherosclerosis (42). Another meta-analysis including 20 studies of patients with psoriasis, with or without PsA, also demonstrated that patients with psoriasis have an excessive risk of subclinical atherosclerosis as evidenced by a higher cIMT and an impaired FMD (43). In this study, an association between psoriasis and CAC was supported. Compared to atherosclerotic markers from peripheral arteries (such as cIMT and FMD), CAC is closely related to atherosclerotic changes in the coronary arteries, which may be directly related to the risk of CAD (44). Therefore, the findings of the current meta-analysis suggest that CAC represents an important pathological factor that increases cardiovascular risk in patients with psoriasis. Our study has several methodological strengths. First, we conducted an updated literature search of three important electronic databases, providing a contemporary assessment of the association between psoriasis and overall CAC. In addition, potential conventional cardiovascular risk factors were adjusted or matched between cases and controls in all of the included studies. Therefore, the association between psoriasis and CAC could be an independent risk factor. Additionally, we also included sensitivity and subgroup analyses, which further support the robustness of the findings. Finally, in addition to the outcome of overall CAC, our meta-analysis suggested that psoriasis is also associated with moderate-to-severe and severe CAC. These findings are important because a recent study indicated that the risk of atherosclerotic cardiovascular disease increased dramatically in people with a CAC score >100 (moderate-to-severe CAC) (45).

Currently, the underlying mechanisms for the association between psoriasis and CAC remain to be fully determined. Vascular inflammation and the interleukin-17a related pathway have been suggested to play a key role according to the recent preclinical studies (46, 47). Overall, inflammation has been recognized as one of the most important mechanisms underlying the pathogenesis of atherosclerosis, including CAC (48). Inflammatory cells and other factors play significant roles in atherosclerosis and in other chronic inflammatory diseases, such as psoriasis (49). Multiple factors can cause abnormal responses in the vascular wall, resulting in inflammation, degeneration, exudation, and hyperplasia (50). Many pathophysiological cellular processes and signaling pathways have been shown to be involved in the development and progression of atherosclerosis, such as recruitment of inflammatory cells, cell proliferation, tissue sclerosis, and neovascularization, as well as activation of the Toll-like receptor 4, the transcription factors nuclear factor-κB, and the Janus kinase (JAK) signal transducer and activator of transcription (STAT) signaling pathways (51). Furthermore, accumulating evidence suggests that anti-inflammatory treatments could be effective strategies for patients with atherosclerotic diseases. Specifically, Canakinumab [Canakinumab Anti-Inflammatory Thrombosis Outcomes Study (CANTOS)] (52) and low-dose colchicine [COLchicine Cardiovascular Outcomes Trial (COLCOT)] (53) have shown promising therapeutic benefits. These findings suggest that inflammation is a key mediator underling the association between psoriasis and CAC. Further investigation is warranted to understand the molecular pathways involved in this association.

Results of the subgroup analysis showed a stronger association between psoriasis and CAC in younger patients (<50 years) compared to the older patients (≥50 years). The *I*^2^ for the overall meta-analysis was 57%, which was reduced to eight and 35% in the subgroup analyses according to age. These findings suggest that age may be a potential source of heterogeneity. These findings are consistent with previous findings which showed that impaired FMD was more pronounced in younger psoriatic patients (mean age <45 years) compared to older patients (≥40 years) (43). Interestingly, an early cohort study showed that, compared to people without psoriasis, the risk of myocardial infarction was increased only in patients with severe psoriasis aged <50 years, but not for those aged ≥50 years (54). Collectively, these findings suggest that psoriasis adversely affects CAC, resulting in more remarkable atherosclerosis in younger patients. While additional prospective clinical studies are needed to validate these findings, our analysis highlights the importance of early cardiovascular evaluation and intervention in younger patients with psoriasis. Older patients with psoriasis are likely to suffer from multiple comorbidities related to cardiovascular diseases, such as hypertension, diabetes, and dyslipidemia, minimizing the relative adverse influence attributed solely to psoriasis on cardiovascular health.

There are several limitations of this meta-analysis that should be noted. First, all of the included studies were case-control and cross-sectional studies. Prospective studies are needed to determine if psoriasis is an independent risk factor of CAC. In addition, because only observational studies were included, a causative relationship between psoriasis and CAC should not be derived solely on the basis of our meta-analysis. For example, the use of biological disease-modifying antirheumatic drugs (bDMARDs) has been associated with reduced risks of cardiovascular events, probably *via* reducing systemic inflammation (55). Therefore, psoriasis patients using these biological therapies may have lower risk of CAC. In addition, use of cyclosporine may increase the risk of dyslipidemia and hypertension, which could increase the risk of atherosclerosis, including CAC (56). Clinical studies should be considered to determine the influence of anti-psoriatic treatment on CAC. Second, extending our literature search to include more electronic databases such as Scopus or Web of Science would strengthen the meta-analysis power. It should be noted that this meta-analysis has not yet been registered. Third, although conventional cardiovascular risk factors were largely controlled among the included studies, we could not exclude the potential existence of residual factors that may confound the association, such as the use of statins. However, a recent meta-analysis failed to show that statins could favorably affect CAC, as determined by the degree of atherosclerosis in asymptomatic populations at high risk of cardiovascular diseases (57). Future studies should evaluate the influence of statins on CAC in patients with psoriasis. Finally, affective disorders such as depression have been established as risk factors for CVD (58) and a predictor of poor prognosis in patients with CVD (59), suggesting an adverse influence of depression on the pathogenesis and progression of atherosclerosis. Interestingly, accumulating evidence suggests that psoriasis patients are 1.5 times more likely to show depressive symptoms than individuals without psoriasis (60), which supports the hypothesis that depression may be an underlying factor in the association of psoriasis and atherosclerosis. Studies are needed to determine the role of depression and the use of antidepressants on the association between psoriasis and CAC.

In conclusion, the findings of this meta-analysis suggest that psoriasis is associated with CAC, and the association may be stronger in younger patients. Studies are needed to determine the mechanisms underlying the association between psoriasis and CAC, and to explore whether interventions against CAC could improve cardiovascular outcomes in patients with psoriasis.

## Data availability statement

The original contributions presented in this study are included in the article/supplementary material, further inquiries can be directed to the corresponding authors.

## Author contributions

YZ designed the study and drafted the manuscript. ZL and LS performed database search, data collection, and study quality evaluation. HW performed statistical analysis. JL interpreted the results. DL revised the manuscript. All authors read and approved the final version of the manuscript.

## Abbreviations

AS: Agatston’s Score
CAD: coronary artery disease
CAC: coronary artery calcification
CT: computed tomography
CI: confidence interval
cIMT: carotid artery intima-media thickness
NOS: Newcastle-Ottawa Scale
FMD: flow-mediated dilation
PASI: Psoriasis Area and Severity Index
OR: odds ratio
PsA: psoriatic arthritis.
